# Supplementary material for: Ana1/CEP295 is an essential player in the centrosome maintenance program regulated by Polo kinase and the PCM
Source: EMBO Rep. 2024 Jan 10;25(1):11. doi: 10.1038/s44319-023-00020-6 (PMC10897187; doi:10.1038/s44319-023-00020-6)
Supplement: Supplementary file 2 — Table EV1 [file 44319_2023_20_MOESM2_ESM.docx]

**Table EV1. List of primers used for dsRNA synthesis**

| **Name** | **CG No.** | **Sequence (5’-3’)** |
| --- | --- | --- |
| ***mCherry* dsRNA** | **-** | **Fw:** TAATACGACTCACTATAGGGATGGTGAGCAAGGG  **Rev:** TAATACGACTCACTATAGGGGTTGACGTTGTAGG |
| ***ASL* dsRNA** | **2919** | **Fw:** TAATACGACTCACTATAGGGAGATTATGGTGAATGCCTTCGAC  **Rev:** TAATACGACTCACTATAGGGAGACTAGCTCAGCCTGCATGATG |
| ***SPD2* dsRNA** | **17286** | **Fw:** TAATACGACTCACTATAGGGAGAGTCGCGTTCCAGCCAAGCAAAGA  **Rev:** AATACGACTCACTATAGGGAGAAATCCCCCACCTCCGTTAAGACTCAG |
| ***D-PLP* dsRNA** | **33957** | **Fw:** TAATACGACTCACTATAGGGAGAGGAGCGCCTAAAGAACAGTG  **Rev:** TAATACGACTCACTATAGGGAGACTGATCGAGCTGTTTGTGGA |
| ***CNN* dsRNA** | **4832** | **Fw:** TAATACGACTCACTATAGGGAGAACCTCCAGGCGGCGGCAACT  **Rev:** TAATACGACTCACTATAGGGAGATGGCTCGAGCGGCATCCTT |
| ***ANA1* CDS dsRNA** | **6631** | **Fw:** TAATACGACTCACTATAGGGAGAATGGCTCTGCAGCTAACAGTAA  **Rev:** TAATACGACTCACTATAGGGAGATTGACCAAAACATGCTCACGCC |
| ***ANA2* dsRNA** | **8262** | **Fw:** TAATACGACTCACTATAGGGAGAATGTTTGTTCCCGAAACGGAGGA  **Rev:** TAATACGACTCACTATAGGGAGATGCTGGGAGCGGTGCGAGGA |
| ***BLD10* dsRNA** | **17081** | **Fw:**TAATACGACTCACTATAGGGAGAACCACCACAACGACCAAA  **Rev:** TAATACGACTCACTATAGGGAGAGATCCTTTCCCTTCTTCTT |
| ***CP110* dsRNA** | **14617** | **Fw:** TAATACGACTCACTATAGGGAGAAAGAAGCGCGAGGTGCAGCT  **Rev:** TAATACGACTCACTATAGGGAGAATGCGATTATGCCGCCTTGG |
| ***CEP97* dsRNA** | **3980** | **Fw:** TAATACGACTCACTATAGGGAGATGTTAAGTCTTCCACCATCGC  **Rev:**TAATACGACTCACTATAGGGAGAGATATGCTACTTACGAAGGCCC |
| ***PLK4* dsRNA** | **7186** | **Fw:** TAATACGACTCACTATAGGGAGAATACGGGAGGAATTTAAGCAAGTC  **Rev:**TAATACGACTCACTATAGGGAGATTATAACGCGTCGGAAGCAGTCT |
| ***SAS4* dsRNA** | **10061** | **Fw:** TAATACGACTCACTATAGGGAGATCTCGCGGCGCTTAGTCGTT  **Rev:** TAATACGACTCACTATAGGGAGAGGCGCAGGATTGGGAGGTG |
| ***SAS6* dsRNA** | **15524** | **Fw:** TAATACGACTCACTATAGGGAGATGTAGTGCGCATGCTGAAGGAC  **Rev:** TAATACGACTCACTATAGGGAGAGCTGCGCTGCTCGTTTATTTTG |
| ***ANA1* 5’-3’ UTR dsRNA** | **6631** | **Fw:** TAATACGACTCACTATAGGGAGATGTGCCTTGAGTGCGTGCTACTTAC  **Rev:**TAATACGACTCACTATAGGGAGATAAATAATCGTCGGGTTTATTAAAATATAAATAAGTTTC |
