# Supplementary material for: Ana1/CEP295 is an essential player in the centrosome maintenance program regulated by Polo kinase and the PCM
Source: EMBO Rep. 2024 Jan 10;25(1):11. doi: 10.1038/s44319-023-00020-6 (PMC10897187; doi:10.1038/s44319-023-00020-6)
Supplement: Supplementary file 3 — Table EV2 [file 44319_2023_20_MOESM3_ESM.docx]

**Table EV2. Full sequence of 5’-3’-UTR-3’UTR hybrid as template for dsRNA**

| **TAATACGACTCACTATAGGGAGA**TGTGCCTTGAGTGCGTGCTACTTACCAGCTGGTATATTTTAGACGCATGTAAATTCTAGTACATTCAATTATTCATCTACGGTCACACTGCCGCTTGGGAGGAATTTTTAAAGACGTTGGGTTGTTTGATTTTACGCTCAAACTTGTTTCGATTTCTACTGCGTAAATGCTGCCCCACATACGAATTTATTACATATATCGATAGAGCAGTCGCCGAACTTTTAATTCGTTTGTTTAGGTTTTAGATTATATTATCCATTTTATGACAATTATTTATATTTTACTTACTTTGCAATTTTGTGTCAAAAAATGACTATCGAAAAAGATTGTATAAAATTTACTCAATAAGTTAAATGTACAATTTTATTACCAATTTGTGTGAAACTTATTTATATTTTAATAAACCCGACGATTATTTAT**TCTCCCTATAGTGAGTCGTATTA** |
| --- |
