# Supplementary material for: Ana1/CEP295 is an essential player in the centrosome maintenance program regulated by Polo kinase and the PCM
Source: EMBO Rep. 2024 Jan 10;25(1):11. doi: 10.1038/s44319-023-00020-6 (PMC10897187; doi:10.1038/s44319-023-00020-6)
Supplement: Supplementary file 4 — Table EV3 [file 44319_2023_20_MOESM4_ESM.docx]

**Table EV3. List of primers used for generation of GFP-nanoPACT construct**

| **GFP_nanobody** | **FW:** GGGGACAAGTTTGTACAAAAAAGCAGGCTTCATGGCGCAGGTTCAGCTGGT  **REV:** GGTACCGCCGCCAGGCCTGCCGGAGCCGCCGCCGCCGGAGCCGCCTTTGCTGCTAACGGTAACCTGGGT |
| --- | --- |
| **StuI_PACT_Fw** | AGGCCTTTCGTGGGCGAACGTATTGCTC |
| **PACT_Stop_attB_Rev** | GGTACCTTTAAGACCCAGCTTTCTTGTACAAAGTGGTCCCCTTACTGATGCCGCGCATGCG |
