# Supplementary material for: Ana1/CEP295 is an essential player in the centrosome maintenance program regulated by Polo kinase and the PCM
Source: EMBO Rep. 2024 Jan 10;25(1):11. doi: 10.1038/s44319-023-00020-6 (PMC10897187; doi:10.1038/s44319-023-00020-6)
Supplement: Supplementary file 12 — Expanded View Figures [file 44319_2023_20_MOESM12_ESM.pdf]

Expanded View Figures

Figure EV1. Candidate screen in *Drosophila* cultured cells for centrosome maintenance.

(**A**) Schematic representation of the different proteins that were depleted in each of the centrosome modules tested for maintenance. These include: "ALL PCM" proteins (simultaneous depletion of four major PCM proteins: ASL, CNN, D-PLP and SPD-2); centriole cap proteins (CEP97 and CP110); the major regulator of centriole biogenesis, PLK4; cartwheel proteins (ANA2 and SAS6) and the centriolar wall proteins (BLD10, SAS4 and ANA1). (**B–F**) Centriolar numbers were assessed considering the positive staining in each cell for different centrosome markers. These include: the PCM marker D-PLP (orange bars); the centriole wall markers SAS4 (green bars), BLD10 (dark green bars) and ANA1 (light green bars) and the distal cap protein CP110 marker (blue bars). Histograms represent the percentage of cells with abnormally low centriole numbers (i.e. 0-1) (**B**) Depletion of "All PCM" (**C**) Depletion of the centriolar cap proteins CP110 or CEP97; (**D**) Depletion of the centriolar biogenesis regulator PLK4, (**E**) Depletion of the cartwheel proteins ANA2 or SAS6, and (**F**) depletion of the centriolar wall proteins BLD10, ANA1, or SAS4. Data information: Bars represent the mean ± SEM of three independent experiments ($n \geq 100$ cells per replicate in each condition). Statistical significance was determined by performing a bimodal regression test. The impact of the different RNAi treatments on the number of cells with 0-1 centrioles was estimated on the number of cells that present a reduced number of centrioles. Estimates indicate the log odds ratio of the indicated treatment on increasing the number of cells with 0-1 centrioles. See Statistical methods for more details. *$p < 0.05$; **$p < 0.01$; ***$p < 0.001$; ****$p < 0.0001$; ns, not significant (see also Fig. 1). Source data are available online for this figure.

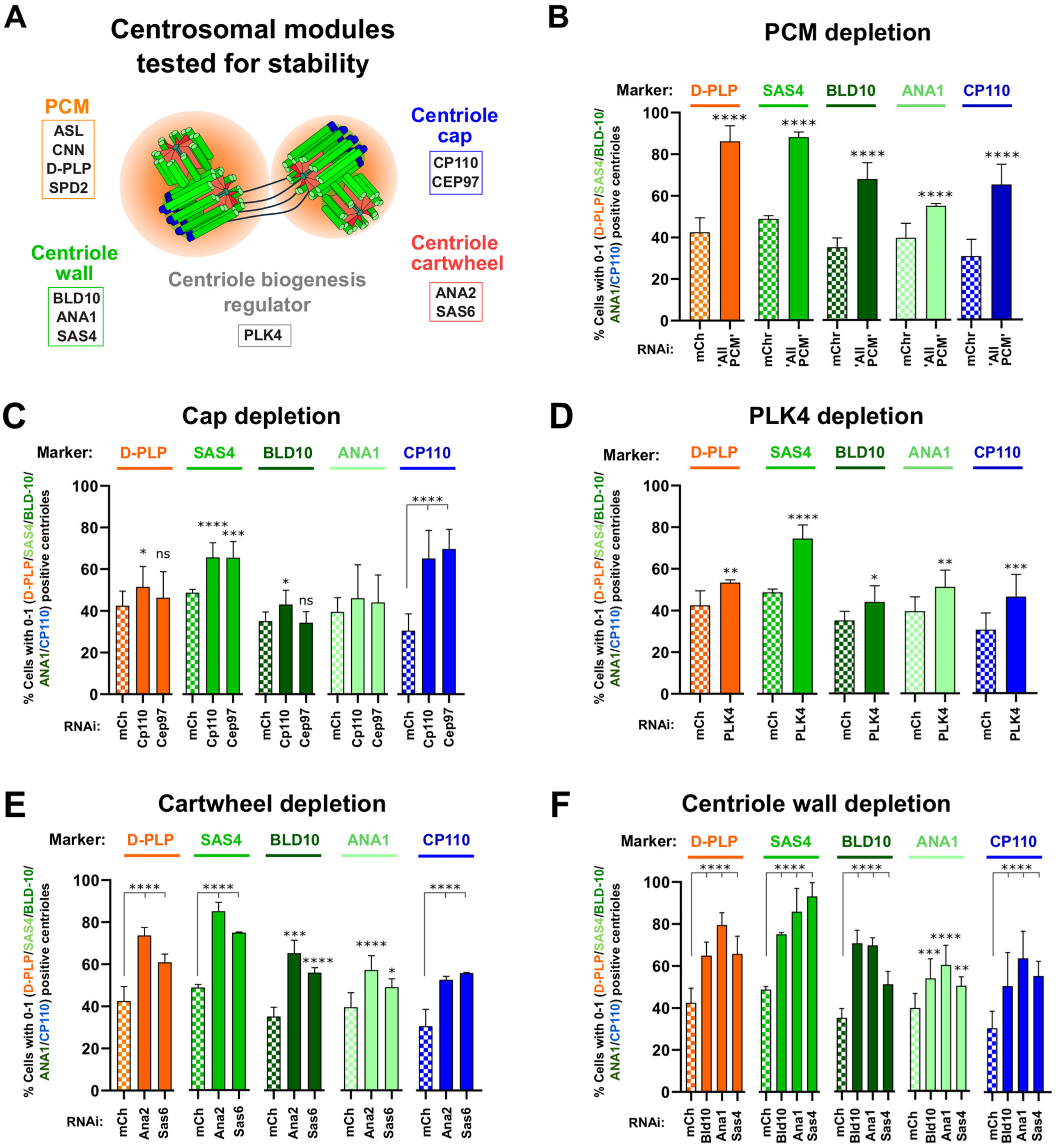
A
Centrosomal modules tested for stability
PCM
ASL
CNN
D-PLP
SPD2
Centriole cap
CP110
CEP97
Centriole wall
BLD10
ANA1
SAS4
Centriole biogenesis regulator
PLK4
Centriole cartwheel
ANA2
SAS6
B
PCM depletion
C
Cap depletion
D
PLK4 depletion
E
Cartwheel depletion
F
Centriole wall depletion
Marker: D-PLP SAS4 BLD10 ANA1 CP110
% Cells with 0-1 (D-PLP/SAS4/BLD-10/ANA1/CP110) positive centrioles
RNAi:
mCh
mChr
'All PCM'
Cp110
Cep97
PLK4
Ana2
Sas6
Bld10
Ana1
Sas4
ns

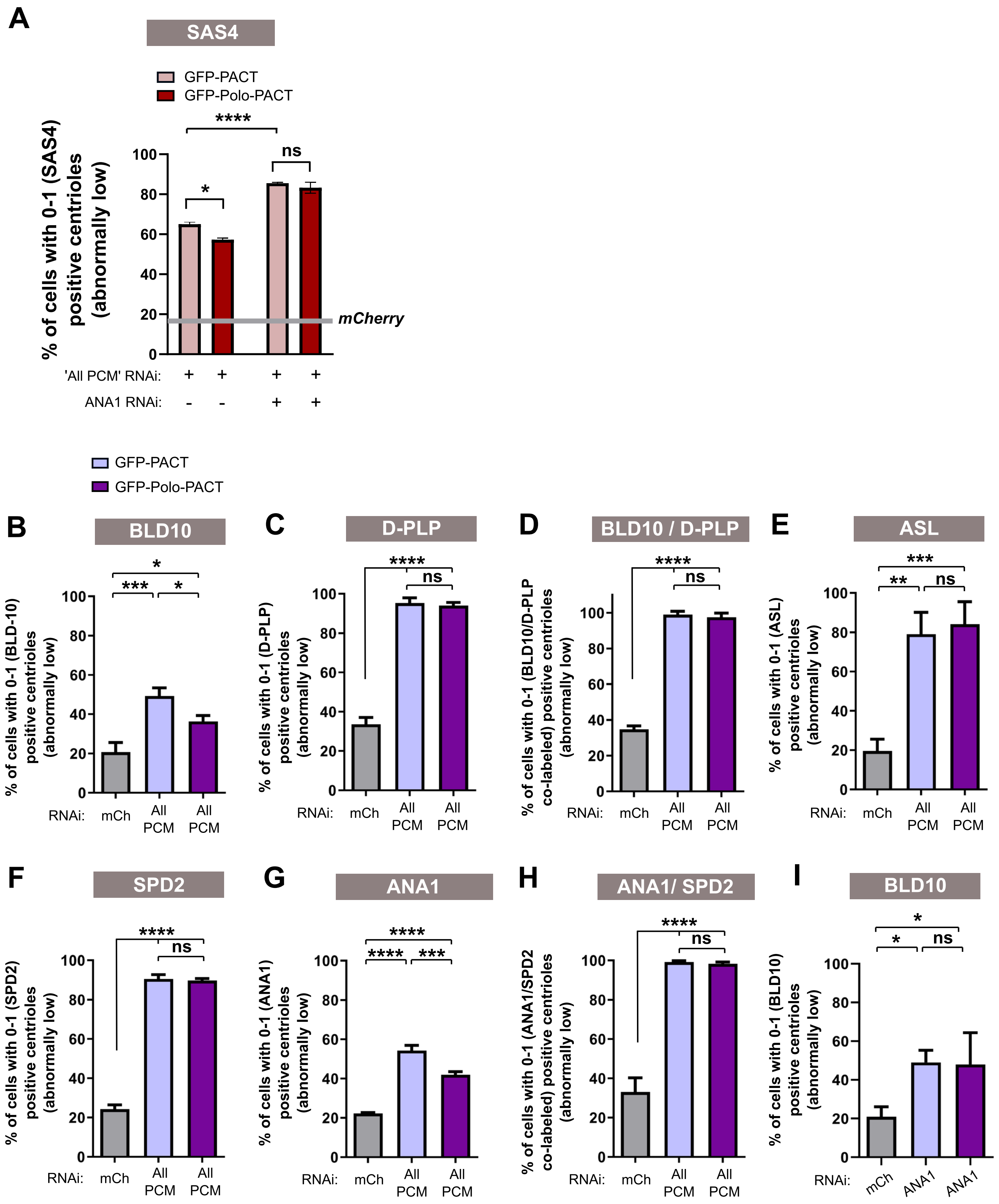
A
SAS4
GFP-PACT
GFP-Polo-PACT
% of cells with 0-1 (SAS4) positive centrioles (abnormally low)
****
ns
*
mCherry
'All PCM' RNAi: + + + +
ANA1 RNAi: - - + +
GFP-PACT
GFP-Polo-PACT
B
BLD10
% of cells with 0-1 (BLD-10) positive centrioles (abnormally low)
RNAi: mCh All PCM All PCM
C
D-PLP
% of cells with 0-1 (D-PLP) positive centrioles (abnormally low)
D
BLD10 / D-PLP
% of cells with 0-1 (BLD10/D-PLP co-labeled) positive centrioles (abnormally low)
E
ASL
% of cells with 0-1 (ASL) positive centrioles (abnormally low)
F
SPD2
% of cells with 0-1 (SPD2) positive centrioles (abnormally low)
G
ANA1
% of cells with 0-1 (ANA1) positive centrioles (abnormally low)
H
ANA1/ SPD2
% of cells with 0-1 (ANA1/SPD2 co-labeled) positive centrioles (abnormally low)
I
BLD10
% of cells with 0-1 (BLD10) positive centrioles (abnormally low)
RNAi: mCh ANA1 ANA1

Figure EV2. Polo kinase induced centriole stability is dependent on ANA1.

(**A–I**) DMEL cells were subjected to dsRNA transfection and treatment with Aph (aphidicolin) and HU (hydroxyurea) at day 0. Cells were harvested and assayed for centriole numbers by immunofluorescence at day 4. (**A**) Depletion of ANA1 prevents Polo-PACT induced centriole rescue, in the context of PCM RNAi. Histogram shows the percentage of cells with abnormally low numbers (i.e. 0-1) of centrioles labelled by SAS4. (**B–H**) Cells were depleted of "All PCM" or mCherry (control). After 16 h, cells were transfected (GFP-PACT or GFP-Polo-PACT) in medium with Aph and HU. Cells were harvested and assayed for centriole numbers by immunofluorescence at day 4. Quantification of the percentage of cells with abnormally low numbers of centrioles (i.e. 0-1). Centrioles were identified by considering the positive staining in each cell for BLD10 (**B**), D-PLP (**C**), ASL (**E**), SPD-2 (**F**) and ANA1 (**G**). Cells with co-staining for BLD10 and D-PLP (**D**) as well as ANA1 and SPD2 (**H**) were also quantified. Note that upon "All PCM" RNAi there is a large increase in the percentage of cells with abnormally low PCM foci per cell (zero or one) in the case of D-PLP, ASL or SPD2, which is not reduced upon GFP-Polo-PACT expression. (**I**) Cells were depleted of ANA1 or mCherry (control). After 16 h, cells were transfected (GFP-PACT or GFP-Polo-PACT) in medium with Aph and HU. Cells were harvested and assayed for centriole numbers by immunofluorescence at day 4. Quantification of the percentage of cells with abnormally low numbers of centrioles (i.e. 0-1). Centrioles were identified by considering the positive staining for the centriolar wall protein BLD10. Data information section: Bars represent the mean ± SEM of three independent biological replicate experiments. For (**A**), $n > 100$ cells per replicate, per condition in each experiment. For (**B–H**) $n > 80$ cells per condition in each experiment. For (**I**) n between 74–128 cells per condition in each experiment. For all the data in this figure, statistical significance was determined by a two-way ANOVA, with Tukey's multiple comparisons test. For all the statistical tests *$p < 0.05$; **$p < 0.01$; ***$p < 0.001$; ****$p < 0.0001$; ns, not statistically significant. Source data are available online for this figure.

© The Author(s)

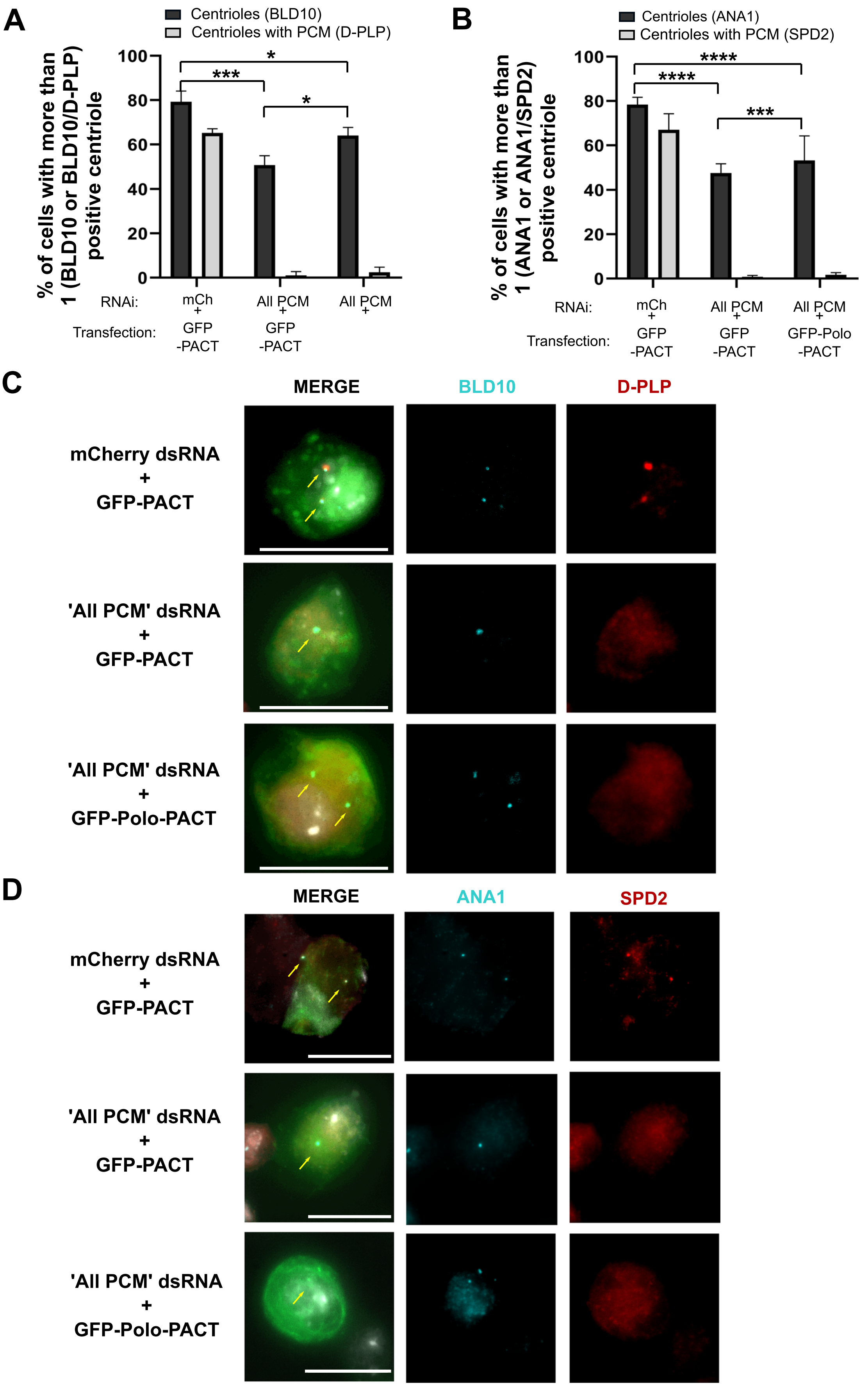

A
Centrioles (BLD10)
Centrioles with PCM (D-PLP)
% of cells with more than 1 (BLD10 or BLD10/D-PLP) positive centriole
100
80
60
40
20
0
***
*
*
RNAi:
Transfection:
mCh + GFP -PACT
All PCM + GFP -PACT
All PCM +
B
Centrioles (ANA1)
Centrioles with PCM (SPD2)
% of cells with more than 1 (ANA1 or ANA1/SPD2) positive centriole
****
****
***
mCh + GFP -PACT
All PCM + GFP -PACT
All PCM + GFP-Polo -PACT
C
MERGE
BLD10
D-PLP
mCherry dsRNA + GFP-PACT
'All PCM' dsRNA + GFP-PACT
'All PCM' dsRNA + GFP-Polo-PACT
D
MERGE
ANA1
SPD2
mCherry dsRNA + GFP-PACT
'All PCM' dsRNA + GFP-PACT
'All PCM' dsRNA + GFP-Polo-PACT

Figure EV3. Rescued centrioles by Polo kinase do not retain PCM markers.

The data in this figure is the same data as in Figure EV2B-D, F-H, analysed in different manner to investigate presence of PCM in centrioles. (**A**,**B**) DMEL cells were subjected to dsRNA transfection and treatment with Aph (aphidicolin) and HU (hydroxyurea) at day 0. Cells were depleted of "All PCM" or mCherry (control). After 16 h, cells were transfected (GFP-PACT or GFP-Polo-PACT) in medium with Aph and HU. Cells were harvested and assayed for centriole numbers by immunofluorescence at day 4. Quantification of the percentage of cells with abnormally low numbers of centrioles (i.e. 0-1). For single markers see Fig. EV2. (**A**) Quantification of the percentage of cells with normal centriole numbers (more than 1 centriole in each cell). Centrioles were quantified by identifying in each cell centrioles positive for BLD10 (dark grey bars), and centrioles which contained PCM, by co-staining for BLD10 and D-PLP (light grey bars). (**B**) Quantification of the percentage of cells with normal centriole numbers (more than 1 centriole in each cell). Centrioles were quantified by identifying in each cell centrioles positive for ANA1 (dark grey bars), and centrioles which contained PCM, by co-staining for ANA1 and SPD2 (light grey bars). (**C**) Representative images of A) are shown. All conditions were acquired with the same exposure. Arrows point to centrosomes in the different cells. MERGE shows the merge of the transfection with the GFP constructs, BLD10, D-PLP and DNA. Scale bar, 10 μm. (**D**) Representative images of **B**) are shown. All conditions were acquired with the same exposure. Arrows point to centrosomes in the different cells. MERGE shows the merge of the transfection with the GFP constructs, ANA1, SPD2 and DNA. Scale bar, 10 μm. Note data while in mCherry controls most centrioles contain PCM, this is not the case after "All PCM" RNAi, even upon GFP-Polo-PACT expression and increase in centriole number. Indeed, in cells depleted of PCM and transfected with GFP-Polo-PACT only 2,5% and 1,7% of cells contain the PCM markers D-PLP (**A**) and SPD2 (**B**), respectively. Data Information: Bars represent the mean ± SEM of three independent biological replicate experiments. For (**A**,**B**) "n">80 cells per condition in each experiment. A Two-way ANOVA, with Tukey's multiple comparisons test was used to test statistical significance. For all the statistical tests used in this figure: $^{*}p < 0.05$; $^{***}p < 0.001$; $^{****}p < 0.0001$. Source data are available online for this figure.

 © The Author(s)

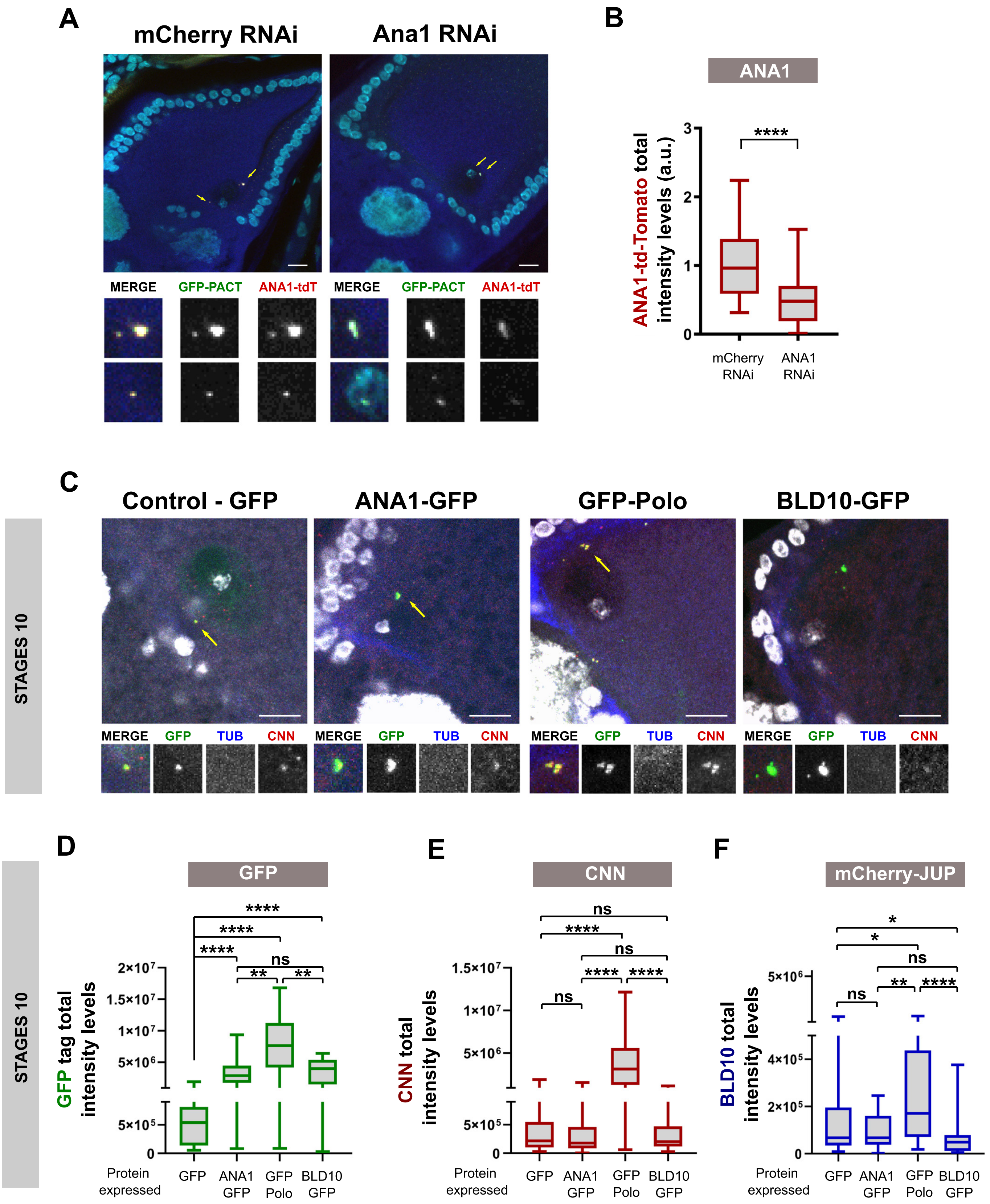
A
mCherry RNAi
Ana1 RNAi
MERGE
GFP-PACT
ANA1-tdT
B
ANA1
ANA1-td-Tomato total intensity levels (a.u.)
****
mCherry RNAi
ANA1 RNAi
C
Control - GFP
ANA1-GFP
GFP-Polo
BLD10-GFP
STAGES 10
MERGE
GFP
TUB
CNN
D
GFP
GFP tag total intensity levels
Protein expressed
GFP
ANA1 GFP
GFP Polo
BLD10 GFP
E
CNN
CNN total intensity levels
F
mCherry-JUP
BLD10 total intensity levels

Figure EV4. Down-regulation of Ana1 by RNAi in oogenesis is not fully efficient and Ectopic tethering of ANA1 to the oocyte centrioles does not increase the PCM protein CNN and microtubule nucleation capacities.

(**A**) Depletion of Ana1 by RNAi. *mCherry*-RNAi (control) and *ANA1*-RNAi were expressed in the germ line using a driver that only expresses after stages 3/4 (i.e., after oocyte specification). Expression of both GFP-PACT [under polyubiquitin promoter; PACT is the centriolar targeting domain of PLP] and ANA1-tdTomato (under endogenous promoter) were used as robust centriolar markers. ANA1-tdTomato was used as a readout to address the efficiency of the RNAi in the depletion of ANA1 protein. Enlargements of the indicated areas (yellow arrows) are shown. All images were acquired with the same exposure. Scale bars, 10 μm. (**B**) Quantification of total intensity levels of ANA1-tdTomato in stage 10 egg chambers for *mCherry*-RNAi and *ANA1*-RNAi expressing oocytes. Box-and-whisker plot (2.5th and 97.5th percentiles) of the total integrated intensity of Ana1-tdTomato. (**C**) Representative images of the analysis of stage 10 oocytes upon tethering different centrosomal proteins to the oocyte centrioles by expressing a GFP nanobody construct fused to the PACT domain (PACT::vhhGFP4) that targets molecules to the centriole. Enlargements of the indicated areas (with centrioles, yellow arrows) are shown. Note that at this stage the oocyte is supposed to have ~64 clustered centrioles, known to be scattered when Polo-PACT is expressed (Pimenta-Marques et al, 2020). Scale bars, 10 μm. (**D**) Quantification of the total intensities of GFP, ANA1-GFP, GFP-Polo and Bld10-GFP tethered to centrioles by PACT::vhhGFP4 in stages 10. (**E**,**F**) Quantification of the total intensities of (**C**) CNN and (**D**) mCherry-Jupiter (a proxi for microtubules as Jupiter is a MAP (Lowe et al, 2014) in stage 10 egg chambers expressing either GFP, ANA1-GFP, GFP-Polo or Bld10-GFP in combination with PACT::vhhGFP4. Note that tethering GFP-Polo leads to an increase in the total levels of CNN and mCherry-Jupiter to the oocyte centrioles in stages 10, which is not observed upon forced localization of ANA1 to the oocyte centrioles. Data Information: For (**B**), $n = 30$ for *mCherry*-RNAi, box minimum = 0.3129, box maxima = 2.240, box median = 0.9621, box 25% percentile= 0.5877, box 75% percentile = 1.388. For ANA1-RNAi, $n = 31$, box minimum = 1.528, box maximum = 1.528 box median = 0.4783, box 25% percentile = 0.1884, box 75% percentile = 0.7012. Box-and-whisker plot (2.5th and 97.5th percentiles) of the total integrated intensity of Ana1-tdTomato. Statistical significance was tested by performing a Unpaired Mann-Whitney test; ****$p < 0.0001$. For (**D**,**E**) Box-and-whisker plots (whiskers extend to the 2.5th and 97.5th percentiles) of the total integrated intensities of the different markers analysed. For (**D**), $n = 31$ for GFP + GFPnanoPACT, box minimum = 54,072, box maxima = 1,887,836, box median = 537,902, box 25% percentile = 139,239, box 75% percentile = 812,266; for ANA1-GFP + GFPnanoPCT, $n = 30$, box minimum = 85,748, box maxima = 9,350,192, box median = 2,850,538, box 25% percentile = 1,727,802, box 75% percentile = 4,443,137; for GFP-Polo + GFPnanoPACT, $n = 26$, box minimum = 87,484, box maxima = 16,806,005, box median = 7,631,831, box 25% percentile = 4,156,380, box 75% percentile = 11,234,152. for BLD10-GFP + GFPnanoPACT, $n = 26$, box minimum= 30,420, box maxima = 6,405,887, box median = 3,945,207, box 25% percentile = 1,477,427, box 75% percentile = 5,383,259. For (**E**), $n = 31$ for GFP + GFPnanoPACT, box minimum = 30,687, box maxima = 1,868,551, box median = 216,522, box 25% percentile = 103,936, box 75% percentile = 546,986. for ANA1-GFP + GFPnanoPACT, $n = 30$, box minimum = 8052, box maximum = 1,525,037, box median = 180,856, box 25% percentile = 86600, box 75% percentile = 460,606; for GFP-Polo + GFPnanoPACT, $n = 26$, box minimum = 786,335, box maxima = 49,501,547, box median = 6,598,892, box 25% percentile = 3,098,434, box 75% percentile = 12,625,516; for BLD10-GFP + GFPnanoPACT, $n = 26$, box minimum = 28,468, box maxima = 1,132,352, box median = 204,727, box 25% percentile = 120,895, box 75% percentile = 472,851. For (**F**), $n = 31$ for GFP + GFPnanoPACT, box minimum = 8875, box maxima = 1,015,035, box median = 66,987, box 25% percentile = 35,287, box 75% percentile = 195,555. for ANA1-GFP + GFPnanoPACT, $n = 30$, box minimum = 2516, box maximum = 245,586, box median = 67,146, box 25% percentile = 38,265, box 75% percentile = 160,067; for GFP-Polo + GFPnanoPACT, $n = 26$, box minimum = 18,307, box maxima = 1,071,604, box median = 170,734, box 25% percentile = 71,301, box 75% percentile = 437,557; for BLD10-GFP + GFPnanoPACT, $n = 26$, box minimum = 4708, box maxima = 376,286, box median = 48,386, box 25% percentile = 12,032, box 75% percentile = 78,575. 3 independent biological replicates were performed for each condition. significance was determined by performing a bimodal regression test. For statistical tests used in this figure: *$p < 0.05$; **$p < 0.001$; ****$p < 0.0001$; ns, not statistically significant. Source data are available online for this figure.

 © The Author(s)

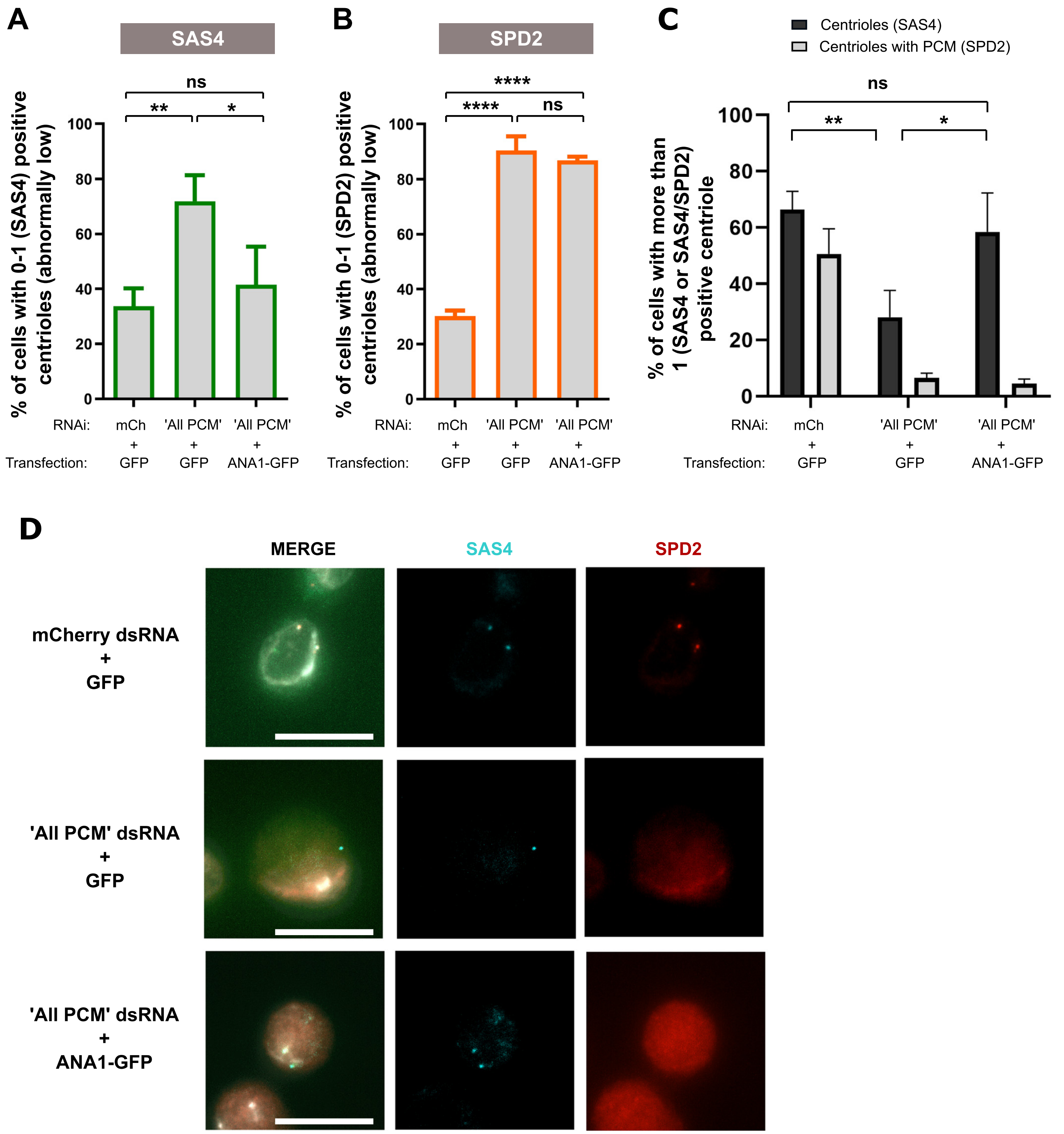
A
SAS4
ns
**
*
% of cells with 0-1 (SAS4) positive centrioles (abnormally low)
100
80
60
40
20
0
RNAi: mCh 'All PCM' 'All PCM'
+ + +
Transfection: GFP GFP ANA1-GFP
B
SPD2
****
****
ns
% of cells with 0-1 (SPD2) positive centrioles (abnormally low)
C
Centrioles (SAS4)
Centrioles with PCM (SPD2)
% of cells with more than 1 (SAS4 or SAS4/SPD2) positive centriole
D
MERGE
SAS4
SPD2
mCherry dsRNA
+
GFP
'All PCM' dsRNA
+
GFP
'All PCM' dsRNA
+
ANA1-GFP

Figure EV5. ANA1 rescues the loss of centrioles induced by PCM depletion.

(**A–D**) DMEL cells were subjected to dsRNA transfection and treatment with Aph (aphidicolin) and HU (hydroxyurea) at day 0. Cells were depleted of "ALL PCM" or mCherry (control). After 16 h, cells were transfected (GFP or ANA1-GFP) in medium with Aph and HU. Cells were harvested and assayed for centriole numbers by immunofluorescence at day 4. Centrioles were identified by considering the positive staining in each cell for the centriolar wall protein SAS4 and presence of PCM was investigated using the marker, SPD2. (**A**) Quantification of the percentage of cells with abnormally low numbers of centrioles (i.e. 0-1) labelled by SAS4. (**B**) Quantification of the percentage of cells with abnormally low numbers of foci with PCM marker SPD2 (i.e. 0-1). (**C**) Quantification of the percentage of cells with more than 1 centriole in each cell. Centrioles were quantified by identifying in each cell centrioles positive for SAS4 (dark grey bars), and centrioles which contained PCM, by co-staining for SAS4 and SPD2 (light grey bars). (**D**) Representative images of (**A–C**) are shown. All conditions were acquired with the same exposure. Arrows point to centrosomes in the different cells. MERGE shows the merge of the transfection with the GFP or ANA1-GFP and SAS4, SPD2 and DNA. Scale bar, 10 μm. Data information: Bars represent the mean ± SEM of three independent biological replicates ("n" between 47-100 cells per replicate, per condition). Two-way ANOVA, with Tukey's multiple comparisons test. For all the statistical tests used in this figure: $^{*}p < 0.05$; $^{**}p < 0.001$; $^{****}p < 0.0001$; ns, not statistically significant. Source data are available online for this figure.

© The Author(s)
